# Comprehensive multi-omics profiling identifies novel molecular subtypes of pancreatic ductal adenocarcinoma

**DOI:** 10.1016/j.gendis.2023.101143

**Published:** 2023-10-14

**Authors:** Xing Wang, Jinshou Yang, Bo Ren, Gang Yang, Xiaohong Liu, Ruiling Xiao, Jie Ren, Feihan Zhou, Lei You, Yupei Zhao

**Affiliations:** a Department of General Surgery, Peking Union Medical College Hospital, Peking Union Medical College, Chinese Academy of Medical Sciences, Beijing 100023, China; b Key Laboratory of Research in Pancreatic Tumor, Chinese Academy of Medical Sciences, Beijing 100023, China; c National Science and Technology Key Infrastructure on Translational Medicine in Peking Union Medical College Hospital, Beijing 100023, China

**Keywords:** Molecular characterization, Molecular subtyping, Multi-omics, Pancreatic cancer, Precision medicine

## Abstract

Pancreatic cancer, a highly fatal malignancy, is predicted to rank as the second leading cause of cancer-related death in the next decade. This highlights the urgent need for new insights into personalized diagnosis and treatment. Although molecular subtypes of pancreatic cancer were well established in genomics and transcriptomics, few known molecular classifications are translated to guide clinical strategies and require a paradigm shift. Notably, chronically developing and continuously improving high-throughput technologies and systems serve as an important driving force to further portray the molecular landscape of pancreatic cancer in terms of epigenomics, proteomics, metabonomics, and metagenomics. Therefore, a more comprehensive understanding of molecular classifications at multiple levels using an integrated multi-omics approach holds great promise to exploit more potential therapeutic options. In this review, we recapitulated the molecular spectrum from different omics levels, discussed various subtypes on multi-omics means to move one step forward towards bench-to-beside translation of pancreatic cancer with clinical impact, and proposed some methodological and scientific challenges in store.

## Background

Pancreatic cancer is regarded as one of the most invasive carcinomas, annually claiming roughly 400,000 lives worldwide,^1^ of which pancreatic ductal adenocarcinoma (PDAC) accounts for approximately 90%. Currently, almost no effective treatment modalities are proposed to prolong overall survival except complete surgical resection followed by six-month adjuvant chemotherapy.^2^ However, over 80% of PDAC patients have already progressed to an advanced stage and are unresectable on diagnosis.^3^ Even worse, most patients amenable to surgery may eventually relapse within three years.^4^ Therefore, new strategies are urgently needed to comprehend this disease better.

Currently, two major aspects are considered to contribute to the grim prognosis of PDAC. For one thing, up to 90% of PDAC consists of a rich stromal compartment,^5^ which contributes to therapy resistance. For another, numerous advances have demonstrated substantial and complicated polyclonal/heterogeneous characterization,^6^ including significant spatial variability and distribution with different molecular and pathologic properties, temporally mutual transformation across different subtypes, and some transitional cell populations and molecular classification within a patient,^7^ which all make it highly flexible and context-related, with few fixed, single, broadly applicable molecular biomarker or targeted therapy. To this end, a deep and comprehensive understanding of the intra-tumoral and inter-tumoral spatial and temporal heterogeneity of PDAC is essential.^8^

Previously, large quantities of research on the heterogeneity of PDAC mainly focused on genomics and transcriptomics, but to some degree, these attempts are not effectively translatable to routine clinical practice due to several limitations. With plenty of high-flux techniques and clinical samples available, integrating multi-omics data systematically and holistically seems to be promising to derive new insight into novel molecular subtypes, biological markers, and complex PDAC biology. This article summarized updated findings of integrated large-scale multi-omics analysis and current knowledge of multiple molecular taxonomies of PDAC, highlighted promising clinical prospects, and discussed both potential opportunities and hurdles to be overcome for translating multi-omics findings into clinical practice.

Thus, this is an ideal time to review our current knowledge of PDAC evolution and heterogeneity, gained from the study of preclinical models and patient biospecimens, and to propose a model of PDAC evolution that takes into consideration findings from varied sources, with a particular focus on the genomics of human PDAC.

**Figure 1:**
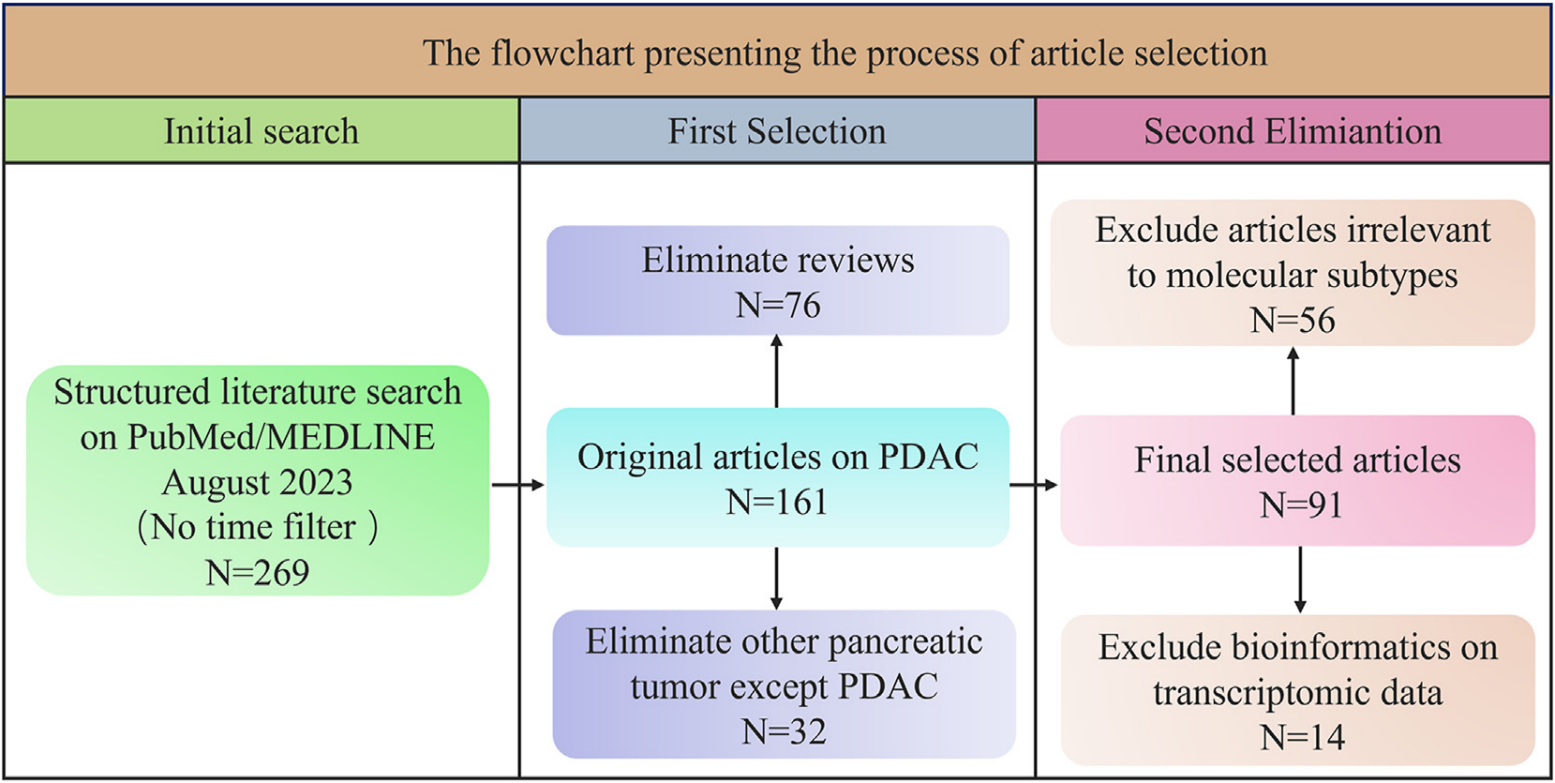
Detailed flowchart of inclusion and exclusion criteria. A structured literature search combining free text words and medical subject headings was used to ensure high recall. After that, repeated siftings were conducted to guarantee highly-correlated articles to be chosen.

To guarantee high accuracy and recall, the structured literature search was conducted on PubMed/MEDLINE in August 2023, combining free text words and medical subject headings (MeSH), followed by manual screening. Keywords were combined using the Boolean operators “AND” and “OR”. No time filter was applied. The full search strategy is displayed as (“Multi-omics”[All Fields] OR “omics”[All Fields] AND (“Pancreatic Neoplasms”[MeSH Terms] OR ((“Pancreas”[Title/Abstract] OR “pancreatic”[Title/Abstract]) AND (“Cancer”[Title/Abstract] OR “tumor”[Title/Abstract] OR “neoplasm”[Title/Abstract] OR “cancers”[Title/Abstract] OR “tumors”[Title/Abstract] OR “neoplasms”[Title/Abstract]))). Based on the initial selection, we put more emphasis on original articles related to PDAC subtyping and further eliminated bioinformatics based on transcriptomics instead of multi-omics. Detailed inclusion and exclusion criteria are shown in Figure 1.

## The molecular profiling of PDAC

**Figure 2:**
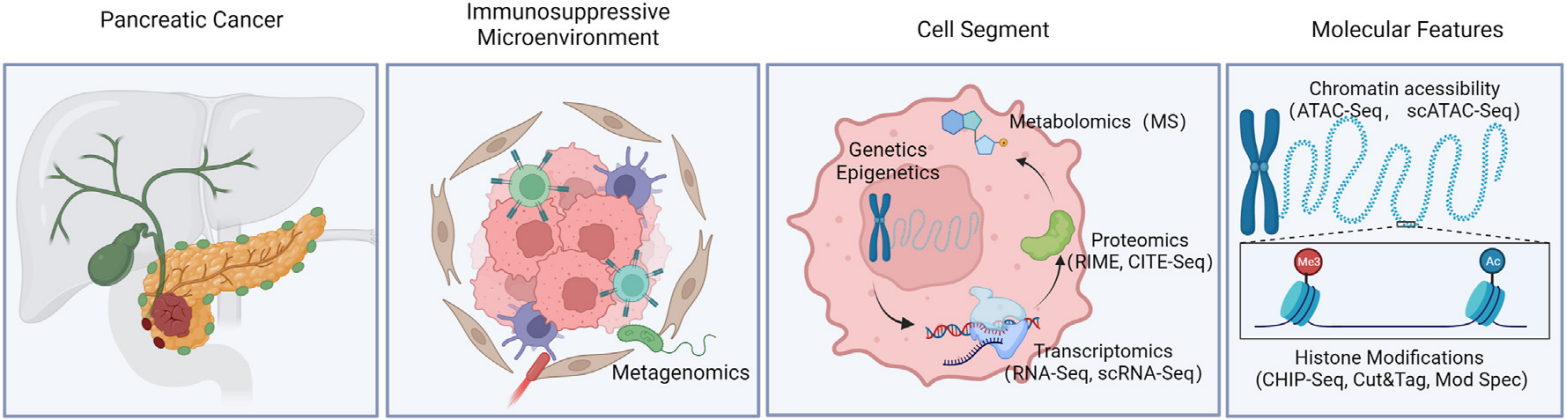
An overview of different dimensions of multi-omics in PDAC. A series of advanced and self-improved multi-omic technologies has enabled the molecular landscape of PDAC to be further depicted and forecasted from macroscopic to microcosmic perspective, from parenchymal to mesenchymal aspect, and from bulk to single cell level. On this basis, the complex molecular subtypes of PDAC are promising to be revealed. Created by Biorender.com. Mod Spec, quantitation of histone PTMs using mass spectrometry; ATAC-seq, assay for targeting accessible chromatin with high-throughput sequencing; CHIP-Seq, chromatin immunoprecipitation sequencing; CUT & Tag, cleavage under targets and tagmentation; MS, mass spectrometry; RIME, rapid immunoprecipitation mass spectrometry of endogenous proteins; Cite-Seq, cellular indexing of transcriptomes and epitopes by sequencing.

Advances in high-throughput technologies have enabled large-scale efforts to analyze multi-omics data in depth including not only the average level of general tissue but also the single cell level, and profile tumors and their complex immunosuppressive environment quantitatively on different levels,^9^ which has laid a solid foundation for an accurate definition of molecular subtypes. An overview of different dimensions of multi-omics and corresponding technology strategies is given in Figure 2 and discussed in detail as follows.

### Genomics

A large amount of previous research focused on the genomic features of PDAC and depicted its relatively comprehensive landscape including germline predisposition genes, somatic mutation, and structural variations.^6^^,^^10^, ^11^, ^12^ With increasing sample set sizes, knowledge and cognition are gradually in-depth, and more mutated genes were identified at a relatively low prevalence of 5%–10%.^6^^,^^13^ When it comes to the classical KRAS mutation which happens in above 90% of cases, current studies showed that it tends to be in the forms of multiple mutations,^14^ and alternative oncogenic driver mutations typically function in *KRAS* wild-type.^14^, ^15^, ^16^

### Transcriptomics

It is acknowledged that screening of differentially expressed transcripts helped to translate and understand the complex PDAC phenotype.^17^ Previous studies have already explored deeply and described in detail the transcriptomic landscape of PDAC, and mainly identified two common subtypes, basal-like and classical, with different survivals.^6^^,^^18^^,^^19^ Besides mRNA, non-coding RNA families play a key role in contributing to various hallmarks of PDAC.^20^^,^^21^ Since the miRNome, lncRNome,^22^^,^^23^ and circRNome^24^ of PDAC had been profiled extensively, many miRNAs and lncRNAs were under investigation for their role as diagnostic, prognostic, or predictive biomarkers and therapeutic targets in PDAC,^25^^,^^26^ such as up-regulated miR-21, miR-23A, miR-155, miR-196, miR-27A,^22^^,^^26^ and hsa_circ_0009065 ^24^ as prognostic markers, and the significantly up-regulated lncRNA — HOTTIP (HOXA terminal transcript antisense RNA) as an attractive therapeutic target.^20^

### Epigenetics

Epigenetic reprogramming, as an emerging hallmark of cancer, is crucial to understanding the oncogenesis and development of PDAC.^27^ For one thing, abnormal DNA methylation pattern affects gene expression in a chromatin state-dependent manner^28^: typically, active promoters are obviously hypomethylated while strong repressive states are significantly methylated, yet at times just the opposite.^29^ Several notable genes silenced by DNA hypermethylation include *BRCA1*, *DNAJC15*,^30^^,^^31^ *MGMT*, *PARP6*,^32^^,^^33^ and *ZFP82*.^34^ Other repressed tumor suppressor genes tend to be in polycomb-repressed or heterochromatin-like states.^29^ For another, histone modification also plays an essential regulatory role, and increased histone deacetylases like histone deacetylase 2/7 were regarded as potential therapeutic targets.^35^ Additionally, critical epigenetic regulators like methyltransferase, deacetylase, and acetyltransferase were markedly activated by enhanced epigenetic states, further epigenetically controlling cancer-related genes from an extensive picture.^29^ Finally, our team recently demonstrated that chromatin 3D structure also serves as an important factor contributing to the heterogeneity and development, especially the metastasis of PDAC.^36^

### Proteomics

In the post-genomic era, proteomic research on tumor cells, tissue, pancreatic juice, blood, or other biofluids from patients has provided a new perspective for facilitating more non-invasive diagnostic and prognostic biomarkers.^17^ Many investigators sampled multiple biofluids from PDAC patients and yielded a series of potential biomarkers. Importantly, LYVE1, REG1A, TFF1 in urine,^37^ apolipoprotein-AII (APOAII) isoforms (especially APOAII-2),^38^ tenascin C isoform (TNC- FNIII-B),^39^ POSTN, and APOL1 LUM^40^ exhibited good diagnostic performance, and overexpressed galectin-1 (LGALS1) in cancer-associated fibroblasts are promising to predict higher metastatic risk and poorer prognosis of PDAC.^41^^,^^42^ Furthermore, phosphor-proteomics could identify both phosphorylated proteins and their phosphor-sites, injecting new vitality into revealing PDAC heterogeneity. Britton et al^43^ identified 152 differentially phosphorylated proteins between PDAC and normal pancreatic tissue,^44^ and Kim et al revealed highly variable phosphor-proteome of varied metastases with different sensitivity to drugs.^45^ In addition, a great number of existing significant findings have not only provided a compelling rationale for biomarker discoveries and glycan-based drugs but also revealed the complicated glycan-based cross-talk of cancer cells and tumor microenvironment (TME).^46^^,^^47^ However, studies on glycoproteomics and glycomics are still in their infancy considering the limitations of analytical techniques and their complex structure including primary chemical structures and varied linkage variability of glycans to a large extent.

### Metabonomics

Metabolic fingerprints serve as an abundant resource to explore more sensitive therapeutic targets or biomarkers from numerous metabolic enzymes and pathways available.^48^ To be specific, bile acids, especially taurocholic acid,^49^ beta-sitosterol, creatine, glycocholic acid, inosine, and sphinganine^50^ in plasma were verified as potential diagnostic biomarkers; lactic acid^51^ and ethanolamine^52^ showed superior performance in differentiating patients with long-term or short-term survivals; both succinic acid and gluconic acid were capable of monitoring the invasion and metastasis of PDAC.^50^ Agents targeting altered metabolism processes like glycolysis, mitochondrial oxidative phosphorylation as well as glutamine and other classical metabolites synthesis are worth further exploring.^53^

### Metagenomics

With the development of metagenomics, quantities of the human gut, oral, and intratumor microbiome are gradually recognized to be potentially associated with PDAC and its risk factors. Pathogenic oral bacteria including *Porphyromonas gingivalis*, *Fusobacterium*, *Neisseria elongate*, and *Streptococcus mitis*, and approximately 10% of *Fusobacterium* in PDAC tissue take part in the carcinogenesis and progression of PDAC. Besides, gut microbial profile by MiSeq sequencing is unique,^54^ and metagenomic classifiers trained on the gut and oral microbiomes could have accurate and specific predicting performance.^55^ Furthermore, combined with metabolomic screening, a series of microbiota-derived metabolites were discovered to play an underlying role in the pathogenesis and treatment response of PDAC like butyrate^56^ and indole-3-acetic acid.^57^

### Molecular subtyping on multi-omics profiles

**Figure 3:**
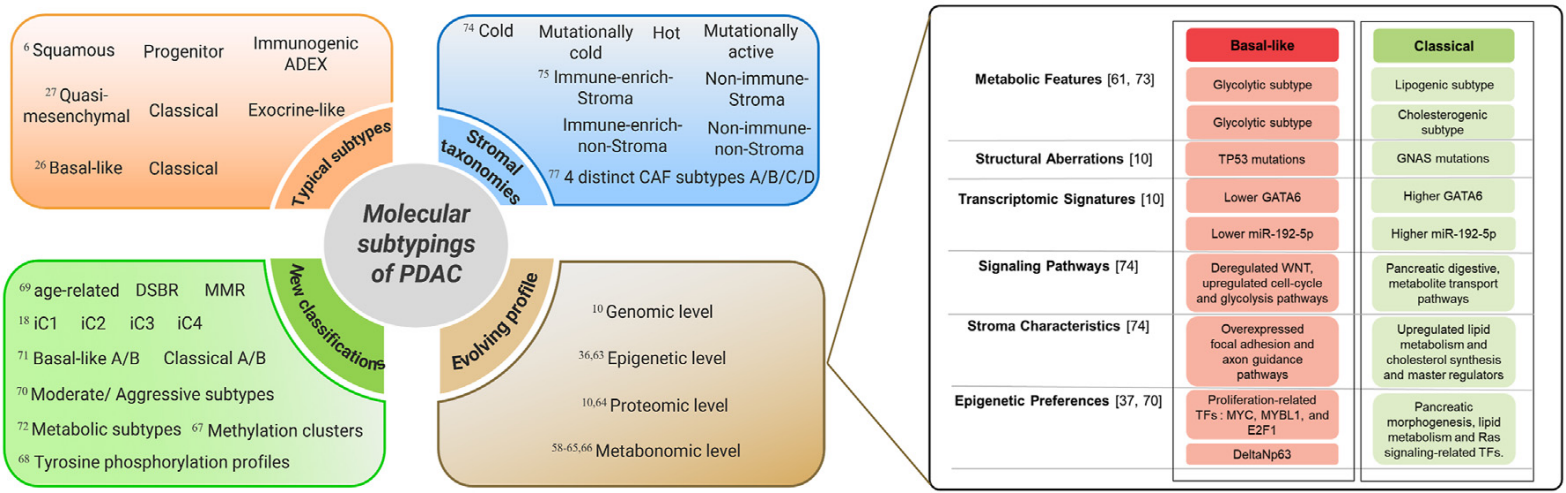
Summary of different subtyping schemas on PDAC. Four main clusters of subtyping schemas including typical transcriptomic subtypes, their evolving molecular profiles, new classifications inconsistent with previous ones, and several taxonomies based on stroma are shown. Each row represents a subtyping schema. The corresponding place in the vertical direction approximates the relationships between them in the part of typical subtypes despite some variability, and the other subtyping schemas are independent of each other. Although a number of multi-omic research reached more or less a consensus on two major transcriptomic subtypes, “basal-like” and “classical”, they enriched each subtype with its evolving multi-dimensional profiles including metabolic features, structural aberrations, transcriptomic signatures, signaling pathways, stroma characteristics, and epigenetic preferences.

Multi-omics analysis of sampled tissue or cell lines has revealed various classification systems on different molecular characteristics of both cancer epithelium and stroma cells. We rearranged and logically illustrated them in this part, as depicted in Figure 3.

### Molecular subtyping concordant with previous ones

Notably, previous studies have validated in primary and metastatic PDAC samples that the squamous (Bailey), quasi-mesenchymal (Collisson), and basal-like (Moffitt) cohorts align well across multiple classifiers, which are resistant and refractory to chemotherapy with a dismal prognosis, which can be distinguished from classical subtype.^6^^,^^18^^,^^19^ Further research efforts on combinations of multi-omics data reached a consensus in varying degrees on two major transcriptomic subtypes, “basal-like” and “classical”, and they further enriched each subtype with its distinct multi-dimensional features from genomic,^14^ epigenomic,^29^^,^^58^ proteomic,^59^ glycomic,^60^ and metabolomic^53^^,^^61^^,^^62^ angels, and provide functional context and forming mechanism to previously identified gene expression subtypes. The detailed progress of each subtype was listed and compared in Figure 3. However, each subtyping still has a wide range of tumor progressive and prognostic features,^59^ suggesting more studies on survival prediction are imperative.

### Novel kinds of molecular subtyping

Besides updating existing classifications, there were also other researchers trying to provide some subclassifications from novel perspectives, which are not mirrored in transcriptomic ones. Considering the great potential of novel kinds of molecular subtyping, we list the detailed study strategies and main discoveries in Table 1.

**Table 1:** New Molecular subtyping and classifiers of PDAC.

Study	Multi-omics involved	Main discovery	Subtypes/classifier
*Novel kinds of molecular subtyping*			
2016 Humphrey^66^ (*n* = 19 cell lines from ATCC series and 17 cell lines from TKCC series)	Mass spectrometry	The third subtype: increased phosphorylation of receptor tyrosine kinases; sensitive to erlotinib	Three subtypes with various sites and forms of tyrosine phosphorylation
2017 Ashton^69^ (*n* = 148 primary PDAC and 12 metastases)	WES and RNA-Seq on bulk biospecimens	MMR: favorable outcomes. DSBR and MMR: increased expression of antitumor immune responses, guiding personalized chemo/immunotherapeutic approaches	Four major subtypes: age-related, DSBR, MMR, and one with unknown etiology
2018 Mishra^63^ (level-3 PDCA data from TCGA)	DNA methylation and RNA-Seq data	Cluster 3: enriched in histological grade G1. Differentially methylated genes of three clusters are mainly aggregated into immune system-related pathways	Three major subgroups with distinct profiles of somatic mutation and CNV
2020 Kong^70^ (*n* = 161 PDAC)	Genomics (CVN and SNV), epigenomics (methylation array), and transcriptomics	iC1: improved prognosis; enhanced genomic stability; higher immune score; lesser CNV	Four clinically related molecular subtypes
2020 Chan-Seng-Yue^72^ (*n* = 330 purified epithelium of primary and metastases from patients)	WES and RNA-Seq	Basal-like-A: major KRAS imbalances; metastases at a late stage; mostly tetraploid. Basal-like-B: minor KRAS disturbances; resectable tumors at an early stage; mainly diploid	Five subtypes including “basal-like-A″, “basal-like-B″, “hybrid”, “classical-A″ and “classical-B″
2021 Ju^71^ (*n* = 146 resectable PDAC patients from TCGA)	mRNA-Seq, mi-RNA-Seq, epigenomics (methylation array), and SNP	KRAS mutation status serves as an essential supplement to MODEL-P subtypes in predicting overall survival	Two prognosis-correlated PDAC subtypes: “aggressive” and “moderate” with different survival outcomes corresponding to DNA damage repair and immune response
2021 Majahan^73^ (*n* = 361 PDAC blood plasma samples)	Plasma metabolome and lipidomics analysis	Subtype 1: decreased level of ceramide; abundant in triacylglycerols. Subtype 2: obvious enrichment of ceramides and sphingomyelin. Subtype 3: reduced sphingolipid metabolites	Three metabolic PDAC subtypes featured by different ranges and profiles of lipid metabolites, especially sphingolipid-related pathways
2021 Espinet^64^ (*n* = 7 FACS-isolated tumor epithelial cells)	Methylation array and transcriptomics	Methylation low/IFN sign high subtype: pro-tumorigenic microenvironment; aggressive; targetable by blocking IFN signaling	Two different origins and etiologies with respective methylation patterns
2022 Rodriguez^60^ (PDAC patients from three public datasets of ICGC, TCGA, and E-MTAB)	Glycomics and transcriptomics	Glyco-code based clusters: O-glycan fucosylated structures are differently expressed which is regulated by GALNT3	Two specific glycan patterns with distinct progressive features, survivals as well as epithelial to mesenchymal transition status
2022 Tong^67^ (*n* = 217 resectable PDAC patients with para-carcinoma samples)	WES, RNA-seq, proteomics, and phosphoproteomics	S-I subgroup: enriched proteins related to glycolysis. S-II subgroup: rich in blood coagulation-related proteins. S-III subgroup: poorest OS. Im-3: higher FH amplicons contribute to lower blood glucose and better prognosis	Three proteome-based subtypes (S-I, S-II, and S-III) with various prognostic features; three immune clusters on immune and stromal cell proportion (Im-1, Im-2, and Im-3)
*Various classifiers of prognosis and therapeutic response*			
2012 Donahue^94^ (*n* = 25 early-stage PDAC)	CNV, mRNA, and miRNA expression	Dysregulated PI3K/AKT pathway and SRC signaling correlated with progression. SRC, PIK3R1/p85α, and CBL are yet to be validated their relationship with PDAC survival	Two prognostic groups are identified on 171 signature genes
2017 Gress^101^ (*n* = 113 FNAB from patients with suspect pancreatic masses)	miRNA and mRNA expression	Molecular algorithms are performed precisely in diagnosing some other solid tumors except cystic lesions	A combined miRNA/mRNA molecular classifier based on SVM has satisfied diagnostic performance
2019 Long^95^ (*n* = 81 PDAC samples)	NGS, transcriptomic meta-analysis, and immunohistochemistry	*ADAM9* and *ANXA2* were compelling therapeutic candidates	A diagnostic and prognostic classifier based on *LAMC2*, *ANXA2*, *ADAM9*, and *APLP2*
2019 Mancera^97^ (*n* = 6 chronic pancreatitis and 19 PDAC)	Chemometric tools and MS.	Differential glycosylation sites of human alpha-acid glycoprotein are identified	Triantennary glycan with two fucoses serves as a potential diagnostic index
2020 Starzyńska^102^ (*n* = 9 PNET, 8 PDAC and 4 INET)	SNV, mRNA, and miRNA transcriptomics	Distinct mRNA/miRNA expression modes in various pancreatic tumors	mRNA and highly-stable miRNAs as effective diagnostic biomarkers of pancreatic tumors
2020 Wang^96^ (*n* = 157 RNA-Seq and 161 CNV of PDAC samples from TCGA)	Gene expression and CNV in the TCGA	A multivariate mixture model (IMIX) was proposed to integrate various types of genomic information and reveal inner correlations	Two groups of good and poor survival based on 104 prognosis-related genes
2021 Xu^12^ (*n* = 171 PDAC samples from TCGA)	Transcriptomics (RNA-Seq), genomics (SNV and CNV)	The 9-gene signature is constructed as a novel prognostic marker to predict the survival of patients and detect the recurrence	An RNA-based risk score prognostic model to evaluate prognostic impact
2021 Yang^103^ (*n* = 66 PDX mouse models of PDAC)	WES, RNA-Seq, miRNA sequencing, and DNA methylation array	*CD55* and *DHTKD1* contributed to intrinsic gemcitabine resistance, and *CDKN1A*, *RRM2*, *EGLN3*, and *PDK1* resulted in the acquired resistance in PDAC. miR-135a-5p was markedly linked with the prognosis and gemcitabine response. Altered PI3K-Akt, p53, and HIF-1 pathways provided candidate target pathways for reducing the acquired resistance	miR-135a-5p for predicting prognosis and gemcitabine response
2021 Yang^104^ (*n* = 176 PDAC from TCGA and Peking 2020 cohort)	Transcriptome and WES analysis	CCL19 serves as a key element to influence the risk-score system constructed by prognosis-associated immune cells	An improved risk-score classifier to identify high-risk groups and guide immunotherapy
2021 Tang^136^ (*n* = 178 tumor and 4 normal samples)	Genomics, epigenomics, and clinical information	A novel wavelet-based model outperformed classic LASSO methods in selecting multi-dimensional variables and considering individual differences	A five-molecule panel based on deep learning method to differentiate high-risk subcohorts from low-risk ones
2023 Wang^99^ (*n* = 178 samples from the TCGA dataset and 84 from the ICGC dataset)	Bulk RNA-seq and scRNA-seq	A constructed ubiquitination-related mRNA-lncRNA model has obvious prognostic advantages over four other known ones	A mRNA-lncRNA-based molecular model with effective prognostic performance
2023 Wang^98^ (*n* = 35 PDAC samples, 34 adjacent tissues, and 31 normal tissues)	Metabolomics and transcriptomics data	An OSPM-related signature consisted of eight genes to tell high-risk from low-risk subgroups with different prognoses and immune features	Three OSPM-related subtypes
2023 Zhou^65^ (*n* = 4 PDAC and 4 pancreatic squamous cell carcinoma)	N6-methyladenine (6^m^A) and 5-methylcytosine (5^m^C) modification array	An outcome-related signature based on seven differentially methylated deficient regions to reflect the prognosis	High-risk and low-risk subtypes on differentially methylated genes

Abbreviations: ATCC, American Type Culture Collection; CNV, copy number variation; PDX, patient-derived xenografts; RNA-Seq, RNA sequencing; scRNA-Seq, single-cell RNA sequencing; SNV, single nucleotide variants; ssGSEA, single sample gene set enrichment analysis; SNP, single nucleotide polymorphism; TKCC, The Kinghorn Cancer Center; GEO, the Gene Expression Omnibus; ICGC, the International Cancer Genome Consortium; TCGA, The Cancer Genome Atlas; WES, whole exome sequencing; FNAB, fine needle aspiration biopsies; SVM, support vector machines; PDX, patient-derived xenografts; PNET, pancreatic neuroendocrine tumors; INET, intestinal neuroendocrine tumors.

Some of these molecular subtypings were performed solely based on DNA methylation,^63^, ^64^, ^65^ tyrosine phosphorylation,^66^, ^67^, ^68^ or glycosylation^60^ which might be exploited deeply for improved patient management. Moreover, a series of new subtyping schemas mainly focused on different states of DNA damage repair and immune response. To be specific, in 2017, Ashton defined four major subtypes based on distinct somatic mutational signatures, among which a double-strand break repair and a mismatch repair group harbored anti-tumor immune activation.^69^ After that, in 2020, the integrated analysis by Kong identified four prognosis-related molecular subgroups and the iC1 subgroup shows better survival outcomes with increased immune scores and lower genomic instability.^70^ Then, Ju et al explored prognostic subtypes of deep learning, and consequently identified “aggressive” and “moderate” subtypes with distinct prognoses, which corresponded to DNA damage repair and immune response.^71^ Notably, the MODEL-P subtype serves as a strong predictor of prognosis, superior to currently most existent practice.

Most well-known subtyping schemes defined molecular subgroups in resectable but not advanced diseases with metastasis which have more aggressive biological behavior and represent a majority of PDAC cases. Recently, a *de novo* reclassification of PDAC from Chan-Seng-Yue revealed that classical and basal-like molecular subclasses exist simultaneously in advanced cases and further supported the essential role of genomic events to form phenotypes of PDAC during progression.^72^ Besides, some studies showed that non-genetic factors, such as chromatin interaction, epigenetic drivers, and transcriptional regulation per se may also exert influence on different phenotypes between primary and metastatic lesions,^36^ well worth investigating further.

For another, most studies were restricted to cell lines, small pieces of resected tumor specimens, or core needle biopsy to define molecular subclasses previously, which could not reveal the whole spectrum of molecular alterations to some extent. Based on 361 PDAC blood plasma samples, Mahajan^73^ clarified three kinds of PDAC programs obviously different in material metabolism including triacylglycerol, ceramide, and sphingolipid generally. It is the first time to determine metabolic subtypes based on easily available blood plasma samples independent of tumor tissue, bringing new energy and ideas.

### Subtypes related to PDAC stroma

The TME which consists of fibroblasts and immune cells provides specific niches for tumor cells to make them amenable to precise immunotherapies and regulates their growth, invasion, and metastasis as shown in Figure 1, but specifically, whether certain stromal or immune elements display as pro- or anti-tumor functions remains largely elusive.^74^ Research revealed a complicated evolution process of subtypes of PDAC stroma as shown in Table 2.

**Table 2:** Updating subtypes based on PDAC stroma.

Study	Multi-omics involved	Main discovery	Subtypes/classifier
2017 Knudsen^75^ (*n* = 109 PDAC cases)	Immunohistochemical analysis and staining and exome sequencing	Different stromal phenotypes: various prognostic impacts; distinct glycolysis-related and hypoxic markers; differentiated immune infiltrations	Four major subtypes: cold, mutationally cold, hot, and mutationally active
2019 Neuzillet^78^ (*n* = 96 PDAC samples)	RNA-Seq	Various CAF classifications could co-exist in a patient. Each CAF subtype has its specific phenotypes including immune-related pathways, proliferative rates, and gene expression profiles, and displays different survival outcomes	Four distinct CAF subtypes
2021 Barbara^77^ (*n* = 32 treatment-naive PDAC resections)	Shotgun proteomics and RNA-Seq	Two kinds of reactive and deserted sub-TMEs exhibit corresponding immune phenotypes and CAF differentiation states. Intra-tumoral sub-TME co-occurrence performs tumor-promoting and chemoprotective functions separately, linking stromal heterogeneity to patient outcome	A prognostic classifier of “TME PHENOtyper"
2021 Tu^79^ (*n* = 23 PDX and FACS-isolated tumor tissue biopsies)	ATAC-Seq, ChIP-Seq and RNA-Seq	Basal-like: regulated by cJUN/AP1; TNF-α-secreting macrophages recruited; pharmacological value of BRD4. Classical: regulated by JUNB/AP1	TNF-α^+^ macrophages regulate phenotypic properties
2022 Wang^76^ (*n* = 269 PDAC samples)	PDAC expression profile data and ssGSEA algorithm	Multiple immune expression patterns, and immune and stromal enrichment molecular markers were discovered	Four subtypes based on different combinations of immune and stromal status
2023 Zheng^81^ (*n* = 11 publicly available datasets from GEO, TCGA, and ICGC)	scRNA-seq and bulk RNA-seq	High-TMGS: more frequent germline mutations and TMB; attenuated immune infiltration but enhanced ICB response rate. Low-TMGS: responsible for chemotherapy and targeted therapy	A constructed TMGS system based on 10 T cell marker genes to predict survival status and treatment response
2023 Wang^80^ (*n* = 5 PMN coupled with tumor-infiltrating immune cells)	Single-cell transcriptomics	TAN-1: poor prognosis. TAN-4: enriched in interferon-stimulated genes	Four distinct TAN subtypes

Abbreviations: ATAC-seq, assay for transposase accessible chromatin with high-throughput sequencing; ChIP-Seq, chromatin immunoprecipitation sequencing; CAF, cancer-associated fibroblast; PMN, peripheral polymorphonuclear leukocytes; TANs, tumor-associated neutrophils; ICB, immune checkpoint blockade therapy.

Dating back to 2017, composite analysis discerned four subtypes of PDAC with different glycolytic and hypoxic biomarkers, and immunological and stromal composition to initially display the heterogeneity of TME.^75^ On this basis, the PDAC cohort is further annotated by Wang et al^76^ into four subtypes on bioinformatic analysis of stromal and immune patterns. Interestingly, based on large-scale integration of histology-guided regional multi-omics, Barbara T. Grünwald revealed two types of “sub-TMEs”, “reactive” and “deserted”, which differ a lot in ECM, CAF activation, and immune features. Different sub-TMEs execute respective tumor-promoting and chemoprotective functions and have an obvious synergistic effect on patient survival.^77^ It allows us to understand the molecular mechanism and annotate stroma classifications on a whole new level.

Moreover, the advancement of single-cell technologies facilitates the dissection and exploration of TME. In detail, CAF subtypes were identified on transcriptomic analysis,^78^ subtype A of which was abundant in “activated stroma” and featured by squamous signature with shorter survival. As two main roles of the innate immune system, TNF-α^+^ macrophages were found to play an essential part in the regulation of phenotypic identity from classical to basal-like subtype^79^; combined analysis of peripheral blood and tumor-infiltrating immune cells depicted the complex landscape of tumor-associated neutrophils, of which terminally differentiated pro-tumor subpopulation tend to have poor prognosis.^80^ For another, a constructed TMGS system based on marker genes of classical cytotoxic T cells helped to predict survival and guide treatment.^81^ Almost certainly, other numerically and functionally impaired immune cells including dendritic cells, and natural killer cells^82^ also play an important part in creating an immunosuppressed TME, whereas how they form their molecular and functional heterogeneity remained unknown. In addition, significant heterogeneity in cellular compositions of TME between primary tumors and metastatic lesions also highlights the critical role of stromal cell constitution in defining advanced PDAC subtypes.^83^

When it comes to the complicated crosstalk between PDAC tumors and TME, Nicolle tended to characterize stroma as basal-like/classical subtype-specific.^62^ The classical subgroup is mainly driven by its surrounding stromal cells and is thus defined as the “classical inflammatory infiltrated” classification.^62^ Correspondingly, in 2018, Puleo et al examined 300 PDAC cases and delineated two subtypes within classical PDAC in high-cellularity samples — the “immune classical” subtype with a markedly vascularized and immune stroma in accord with “classical inflammatory infiltrated” classification, and “pure classical” subtype without immune infiltration.^84^ Only when incorporating all low-cellularity samples, two additional stromal subtypes, “stroma activated” and “desmoplastic”, were discovered, each of which shows characteristics of both classical and basal-like epithelial subtypes, inconsistent with convergent tumor/stromal classifications previously described.^62^ Collectively, it is tough to differentiate whether stromal and epithelial subtyping are mutually overlapped or independent to contribute to the poor prognosis of PDAC.

### Various classifiers of prognosis or therapeutic response

High-throughput multi-dimensional analysis also provides a new paradigm for refining prognosis-related genes, and constructing novel prognostic classifiers, thus stratifying patients into clinical groups correlated to survival more accurately.^92^^,^^93^ Firstly, in 2012, Donahue et al performed a survival-based integrated analysis and identified 171 genes segregating patients into two statistically significant prognostic groups.^94^ Then a random forests model affected by *LAMC2*, *ANXA2*, *ADAM9*, and *APLP2* also exhibited an excellent prognostic performance proposed by Long et al in 2017.^95^ Following this, in 2020, Kong et al identified 35 differentially expressed genes associated with prognosis between iC1 and the others.^70^ After that, in 2020, Wang et al applied a multivariate mixture model (IMIX) to detect 104 genes associated with survival outcomes.^96^ Additionally, in 2021, Xu et al used the Lasso method to ascertain nine characteristic genes and constructed a prognostic signature to reflect the recurrence risk of PDAC.^12^ Also, it should be noted that in 2021 Barbara proposed that stromal heterogeneity, namely co-occurrence of different sub-TMEs, was strongly linked to poor outcomes, and constructed the “TME PHENOtyper” model using 72 genes whose accuracy reached 95.7%.^77^ Notably, besides classical mRNA, key proteins or their certain modifying sites,^67^^,^^97^ metabolites^98^ and mRNA-lncRNA^99^^,^^100^ or mRNA-miRNA^101^^,^^102^ networks all serve as important complementary tools to construct diagnostic and prognostic classification systems.

As for predicting treatment responses, by integrating and comparing multi-omics data before and after gemcitabine treatment, Yang et al revealed the potential of miR-135a-5p to predict the gemcitabine response, the relevance between *CD55* and *DHTKD1* and intrinsic gemcitabine resistance, and the relationship between *CDKN1A*, *RRM2*, *EGLN3*, and *PDK1* and acquired resistance.^103^ Besides, by analysis of transcriptome and whole-exome sequencing, in 2021, Yang et al took CCL19 into predicting model and perfected the risk score of two prognosis-related immune cells, which could predict immunotherapy sensitivity.^104^ To summarize, robust, precise, and practicable biomarker panels are imperative, and novel subtypes and classifiers are systemically depicted in Table 1.

## Guiding value to the clinical practice

### Therapeutic strategies tailored to specific subtypes

During the evolving definition of classifiers and molecular classifications, the widespread perception was produced that a core set of fixed pathways or molecules will prove to be potentially valuable for subtype-specific anticancer therapies. Particularly, it was reported that *KRAS* wild-type PDAC tends to harbor other RAS pathway gene alterations like elevated RTK and mTOR signaling suggesting a potential therapeutic opportunity.^14^ Nicolle et al found inhibiting highly epigenetically deregulated NPC1L1 with Ezetimibe might be an efficient treatment approach and basal-like subtype with lower NPC1L1 was more sensitive than classical subtype.^62^ Two molecular classifications raised by Sinkala et al reminded us that targeted inhibition of RAGE may be effective to more severe subtypes, while less severe ones may be more responsive to inhibitors of ion channels and membrane pump proteins.^59^^,^^88^ Daemen proposed that metabolic vulnerabilities are exploited for cancer therapy, and glycolysis, glutamine metabolism-blocking, or ROS-inducing agents may be particularly effective in mesenchymal tumors.^53^ Tong discovered different kinase and substrate profiles of S-I and S-III, suggesting differentially sensitive therapeutic strategies.^67^ Detailed features of distinct subtypes are listed in Table 1.

### Novel and specific druggable targets

Multiple publications have consistently revealed that approximately 25% of patients may harbor genetic alterations to guide treatment decisions.^85^ Considering the absence of specific druggable genetic alterations at present, mapping of distinct epigenetic marks like super-enhancers and nuclear factors, kinases, or other small molecule proteins with elevated activities, and different non-coding RNAs involved in tumorigenesis and development could help to discover potentially actionable candidates for biomarkers and pharmacological targets.^29^^,^^59^^,^^62^^,^^86^

Notably, Nicolle uncovered epigenetically deregulated pathways including WNT, EGFR, and PPARG in PDAC subtypes with high potential druggable prosperity.^62^ Interestingly, multiple approaches targeting nucleic acids have gained more and more attention, including small interfering RNAs and gene-editing techniques like CRISPR-Cas9. Some studies confirmed miR-21, miR-23A, and miR-27A could inhibit the proliferation of PDAC jointly,^86^ and miR-126 and miR-206 could regulate *ADAM9* and *ANXA2* respectively, and be involved in the metastasis of PDAC.^89^, ^90^, ^91^ Also, KRAS-associated dysregulated regulative networks of non-coding RNAs are identified. Thus, all of these may aid in the development of RNA-based anti-PDAC therapies.^105^

### Treatment protocol enabling the transition of subtypes

Conversion from a highly aggressive to a favorable subtype is also considered an effective strategy of treatment to resist inflamed and aggressive states. Gwen revealed the plasticity between subtypes from the basal-like subtype to the classical identity through inactivation of MET kinase, supporting the prospect of anti-MET therapies in PDAC.^29^^,^^87^ Pharmacological inhibitors of BRD4 contributed to the restoration of the classical subtype with a less severe prognosis.^79^

### Potential therapies targeted at the immune microenvironment

With the widespread use of single-cell RNA-Seq, comprehensive atlases of various immune cells like cytotoxic T cell,^81^ macrophages,^79^ and neutrophils^80^ were depicted to provide clues for immunotherapies which targeted specific subclusters at their metabolic or epigenetic features based on their respectively promotive or inhibitory effects to the development of PDAC.

### Phenomics and drug screening

Phenomics which studied phenotypes under various environmental conditions, once combined with multi-omics, would inject new vitality into drug screening. In the beginning, cell lines served as the cornerstone of drug development given its stability and accessibility. As an unbiased algorithm, SCN rank could systematically and deeply integrate cell-line data, thus promoting multi-omics-based drug ranking and identifying responsive gene signatures.^106^ However, there is still a long way to go considering the obvious gap between cell-line models and real biological tissue.^107^ High-throughput drug screening platforms on patient-derived organoids could solve this problem to some extent, and conditionally reprogrammed cell methodology could further increase the success rate.^108^

### Prospects and challenges

**Figure 4:**
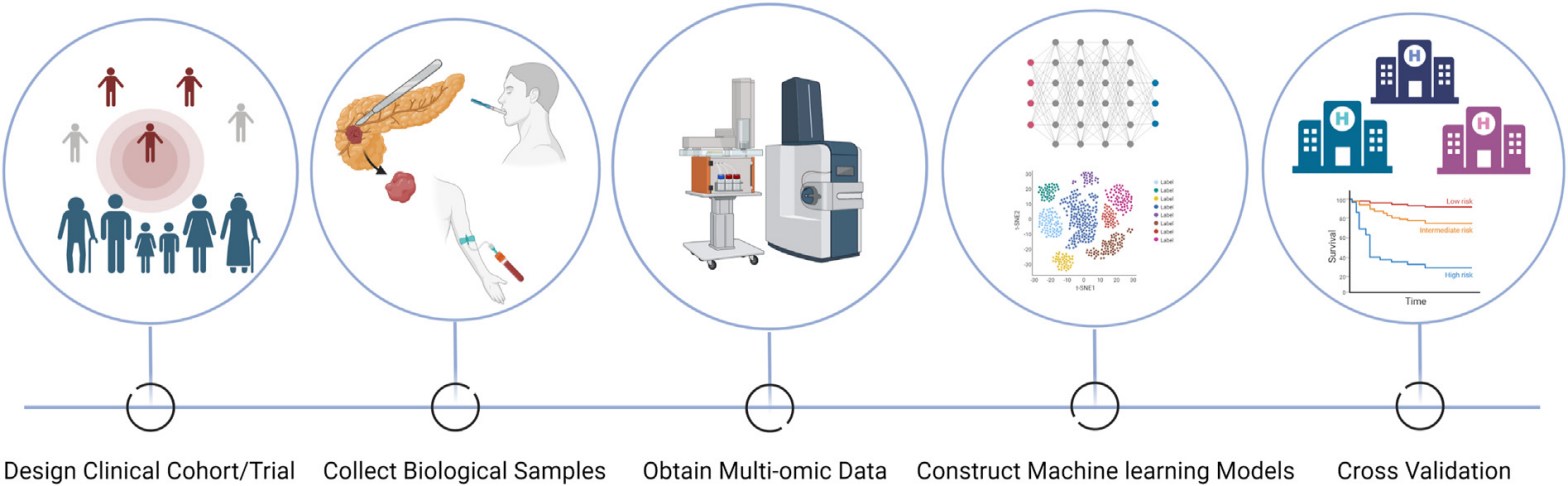
Basic pattern of multi-omics study from clinical needs to finding transformation. Multi-omic studies typically obey the basic mode from the initial clinical cohort/trial design to the final finding transformation and promotion. Only by overcoming challenges during the whole process and mastering the main features of each step can we obtain the desired results. Created by Biorender.com.

Generally, studies based on multi-omic methods and data follow the basic process from the initial design of clinical studies to the final finding transformation as shown in Fig. 4. Throughout the whole process, each step comes with its share of prospects and challenges. Certainly, multi-omic data-based subtypes appear more informative and could reduce confounding and interfering effects of biological, experimental, and statistical noises.^17^^,^^109^^,^^110^ Moreover, with the decrease in cost and continuous development of high-throughput sequencing technology, larger and more diverse omics datasets are available,^111^^,^^112^ enabling more research to launch and more advanced classification schemes of PDAC to construct. However, how to properly integrate multi-omics data from increasingly complicated technical and methodological choices and obtain sufficient evidence to translate clinical use remains a topic worth investigating. Next, we will discuss them in detail based on different stages of the basic process.

### Considerations on acquisition and selection of different omics data

Notably, even nowadays, obtaining full-spectrum multi-omics at single-cell and spatial resolution still has a long way to go. For example, comprehensive and error-free profiling of all genetic variants, and multimodal integration of epigenomic features like histone PTMs, proteome, metabolome, and lipidome assays are all still big challenges.^113^ Besides, instead of transient and static data, ancestral and ever-changing states of live cells contain more valuable information but are obviously more difficult to acquire and analyze.

Even if we could get access to high-dimension and dynamic big data in the decades to come, we still come to draw wrong conclusions easily. Admittedly, big data is featured by highly varied volume, velocity, and valence, but does not necessarily have high veracity and value. A good case in point is that some normal adjacent tissues were taken for certain classifications during the evolution of molecular subtypes of PDAC. Certainly, data has no problem itself, but some subtle perturbation from the process of study will have a huge impact on it and even the final result. Thus, we should put more emphasis on quality control and management, and design more checkpoints during sample obtaining and processing to guarantee the authenticity and reliability of data, or investigate better post-hoc analyzing and integrating methods to mask noise and disturbance.

As for how to choose from complex types of multi-omics, many studies attempted to explore genetic alterations to reveal intra-tumoral heterogeneity and mostly employed a combination of genomics and transcriptomics but lacked other combinations.^114^^,^^115^ Different omics with their features and advantages should be well-exploited as mentioned above. Emerging omics like epi-transcriptome^116^ are promising to inject new vitality. Some other valuable dimensions include clinical information,^117^ pharmacological profiling,^15^ and immune hallmark.^118^ Thus, a comprehensive integration of all varied information from molecules, and microbiome to clinical profiling is necessary for elucidating how they complement and interact with each other to take effect, and meanwhile guarantee the veracity and facticity of data obtained.

### Problems with proper choices of integrating method

Molecular classification of cancer can be approached in many ways, including machine learning and deep learning approaches.^119^ However, it remains technically difficult to effectively leverage and integrate ever-increasing multi-omics data.^120^ The main hurdles include the heterogeneity of experimental samples and analytic protocols, varied data qualities and manifestations, an imbalance between numerous molecular features, and a relatively limited sample size.^59^^,^^121^^,^^122^ Currently, increasing studies put benchmarking and prioritizing diverse methods and tools as priorities. Deep learning-based dimensionality reduction technique, such as variational autoencoder, is a promising approach to the dilemma of unbalanced dimensionality^123^; IMIX combined with summary statistics could realize integrative analysis of continuous numerical or binary variable, time-to-event survival data, and other forms of outcomes.^96^ However, a valid and robust approach to combining omics data mentioned above with common non-omics has yet to be explored further with continuous efforts to tackle these difficulties.

For another, existing algorithms could only discuss and quantify the strength of the relationship between genes within the same batch of data in one dataset. However, due to restrictions on sample quantity, multi-omics data always failed to be obtained from the same patient, even from the same dataset. Thus, approximate distance correlation is of great meaning to solve this problem to some extent and has been proved to precisely specify the relationship across interrelated genes from different sources.^124^ Moreover, a Bulk2Space algorithm based on deep learning could make bulk RNA-seq data, single-cell, and spatial transcriptomics complementary by one another, which especially could be an important supplement to those bulk sequencing without its single cell-level data^125^

### Difficulties in obtaining PDAC samples

Up to now, the phenotypic study of early and late PDAC has been impeded by a lack of sufficient and high-quality surgical samples. One major obstacle can be abundant infiltrating stromal cells which notoriously surround PDAC.^62^^,^^126^ Another barrier can be attributed to the aggressive nature of this disease, making a large proportion of patients inoperable and opportune biopsies problematic. Especially as a series of therapies come to be ineffective, obtaining viable and sufficient tumor samples is increasingly difficult due to treatment-induced changes.

However, analyses of multi-omics data were heavily confounded by the low neoplastic purity and complex different cell types.^83^ Given this, researchers have employed various techniques and strategies to purify tumor samples: some tended to use single-cell technologies to facilitate the dissection of tumor and TME experimentally; some tried to apply some new sampling instruments like next-generation endoscopic ultrasound-guided fine-needle core biopsy needles^127^^,^^128^ or mechanical enrichment techniques like macro-dissection or laser capture microdissection^18^^,^^129^^,^^130^ to increase the quality and quantity of samples; the others combined some computational methodology like blind source separation^131^ or support vector machines^14^ to digitally and virtually separate tumor and helped to achieve clustering results independent of purity. Interestingly, liquid biopsy might provide a potential alternative resource to allow for real-time genomic and epigenomic profiling but for tissue biopsy.^132^^,^^133^ Besides, cancer-derived extracellular vesicles also function as a pivotal role in the interaction between pancreatic normal epithelial cells and cancer cells and could provide more valuable information for multi-omics studies.^134^

### Issues involved in clinical translation and applications

Besides tumor purity and clustering algorithms, sample size and the proportion of resectable and metastatic lesions are also essential elements to affect the accuracy and authenticity of the final classification. However, most studies mainly focus on primary untreated tumors rather than advanced ones which more patients suffer from.^135^ They should be investigated separately considering absolutely different biological features of both epithelium and stoma.^36^^,^^72^^,^^83^ Moreover, most classifications are proposed in theory, but not verified by experiments or clinical practice. Considering the difficulties and limitations of building animal models, further well-designed clinical trials using large cohorts are warranted urgently. In addition, internal or external pressures will continuously motivate molecular composition and profiling to transform during the progression and treatment, also highlighting the need to construct the platform to collect longitudinal and time-varying molecular data in cooperative and multidisciplinary clinical assays thoroughly.^135^

In summary, definitions of molecular subtypes are a progressive course as the classifiers and cut-offs are renewing and refining continuously, and they share tight associations with each other, working together to crystalize the molecular subtyping of PDAC. Only by mastering more accurate and high-quality information from multi-dimension in an easily accessible way and optimizing diverse advantages of rapidly changing technologies, can we make positive progress in better management of PDAC.

## Conclusions

Effective integration of multi-omics data is of vital significance to reveal the complicated molecular landscape of PDAC and point out the further direction toward precision medicine. Ideally, with the advent of multiple high-throughput screening platforms at professional technique and a reasonable cost, a more nuanced analysis of multi-omics of primary lesions and metastases, epithelium, and TME, combined with proper computational tools will further decipher the underlying mechanism and molecular classifications, and produce a therapeutically actionable view. Future work is encouraged to advance into well-designed validation investigation to capitalize on these findings, and both cross-sectional and longitudinal molecular data-sharing platforms under sufficient quality control remain to be established to drive progress forward.

## Author contributions

Study concept and design: X.W., J.Y., B.R., G.Y., X.L., R.X., J.R., F.Z., L.Y., and Y.Z.; Drafting of the manuscript: X.W., J.Y., and B.R.; Critical revision of the manuscript for important intellectual content: L.Y. and Y.Z.; Funding: L.Y. and Y.Z. All authors read and approved the final manuscript.

## Conflict of interests

The authors declare that they have no competing interests.

## Funding

This study was supported by the 10.13039/501100001809National Natural Science Foundation of China (No. 81972321, 82273455to L.Y.), CAMS Innovation Fund for Medical Sciences (CIFMS) (No. 2021-I2M-1-002 to Y.Z.), the National High Level Hospital Clinical Research Funding (China) (No. 2022-PUMCH-D-001), and the National Multidisciplinary Cooperative Diagnosis and Treatment Capacity Building Project for Major Diseases (China).
